# Preoperative Triglyceride-Glucose Index as a Predictor of Weight Loss Following Sleeve Gastrectomy: A Retrospective Cohort Study

**DOI:** 10.1007/s11695-026-08643-z

**Published:** 2026-03-29

**Authors:** Muhammet Fatih Keyif, Ferdi Bolat

**Affiliations:** Bolu Abant İzzet Baysal University, Bolu, Turkey

**Keywords:** TyG Index, Sleeve Gastrectomy, Bariatric Surgery, Weight Loss Prediction, Metabolic Risk

## Abstract

**Background:**

The triglyceride-glucose (TyG) index is a simple and cost-effective surrogate marker of insulin resistance. While its role in predicting cardiometabolic risk is well established, its association with postoperative weight loss in bariatric surgery remains underexplored. This study aimed to evaluate the predictive value of the preoperative TyG index for weight loss outcomes following sleeve gastrectomy.

**Methods:**

In this retrospective cohort study, data from 122 patients who underwent laparoscopic sleeve gastrectomy between January 2019 and January 2024 at a tertiary care center were analyzed. Preoperative TyG index values were calculated using fasting glucose and triglyceride levels. Patients were grouped based on a TyG cut-off value determined by ROC analysis. Associations between the TyG index and postoperative weight loss—both in absolute (kg) and percentage terms—were assessed using Pearson correlation and multivariable linear regression.

**Results:**

The mean TyG index was significantly higher in patients with greater postoperative weight loss (*p* < 0.05). ROC analysis identified a TyG cut-off of 8.7, which yielded a sensitivity of 75.4% and a specificity of 63.9% for predicting significant weight loss (AUC = 0.73). In multivariable analysis, the TyG index remained an independent predictor of postoperative weight loss after adjustment for age, sex, and BMI. Patients with TyG ≥ 8.7 demonstrated significantly higher absolute and percentage weight loss compared to those below this threshold.

**Conclusion:**

The preoperative TyG index is independently associated with postoperative weight loss outcomes in patients undergoing sleeve gastrectomy. Its ease of calculation and accessibility support its potential role as an adjunctive metabolic marker in preoperative counseling and risk stratification, rather than as a standalone tool for surgical decision-making. Prospective, multicenter studies are warranted to validate these findings and to clarify the clinical applicability of TyG-based thresholds.

## Introduction

Obesity has become a major global public health concern due to its rapidly increasing prevalence and strong association with numerous associated medical problems. It is closely linked to type 2 diabetes, hypertension, dyslipidemia, obstructive sleep apnea, and cardiovascular diseases, and is a major contributor to reduced quality of life and increased mortality [1, 2]. Conventional lifestyle interventions and pharmacological treatments often fail to achieve sufficient long-term weight loss, making bariatric surgery a highly effective and increasingly preferred option, particularly in cases of severe obesity [3, 4].

Sleeve gastrectomy, one of the most commonly performed bariatric procedures, has gained popularity due to its efficacy and relatively low complication rates [5, 6]. However, weight loss outcomes following sleeve gastrectomy are highly variable among individuals. This variability underscores the clinical importance of identifying preoperative predictors that may help estimate postoperative weight loss potential. The ability to anticipate postoperative outcomes before intervention may assist preoperative counseling and risk stratification, and support the development of more individualized treatment strategies [7–9].

The triglyceride-glucose (TyG) index has recently emerged as a surrogate marker for insulin resistance and has been associated with various cardiometabolic disorders, including metabolic syndrome and metabolic dysfunction–associated steatotic liver disease (MASLD) [10]. As it relies on routine and inexpensive laboratory parameters—fasting triglyceride and glucose levels—the TyG index offers a practical and cost-effective tool for clinical use [10]. Some large-scale studies have shown a strong association between the TyG index and metabolic dysfunction [11, 12]. Similarly, El-Sehrawy (2025) reported that the TyG index exhibited good discriminatory power in diagnosing metabolic syndrome [13].

Despite growing interest in the TyG index as a metabolic biomarker, its prognostic value in the context of bariatric surgery—particularly for postoperative weight loss following sleeve gastrectomy—remains underexplored. This study aims to evaluate the association between preoperative TyG index levels and weight loss outcomes after sleeve gastrectomy. By focusing on metabolic rather than purely anthropometric predictors, the findings may contribute to improved preoperative assessment and more nuanced clinical counseling, rather than definitive surgical decision-making, in bariatric practice.

## Materials and Methods

### Study Design, Population, and Ethical Considerations

This retrospective, single-center observational cohort study was conducted at the Department of General Surgery, XXX University Faculty of Medicine (XXX, YYY). The electronic medical records of patients who underwent laparoscopic sleeve gastrectomy between January 2019 and January 2024 were reviewed.

A total of 122 patients who met the following inclusion criteria were enrolled: aged between 18 and 65 years, availability of preoperative fasting triglyceride and glucose measurements, and at least 12 months of postoperative follow-up. Patients were excluded if they had type 1 diabetes mellitus, secondary causes of obesity such as endocrine or genetic disorders, or incomplete or inconsistent medical data.

The study protocol was reviewed and approved by the Non-Interventional Clinical Research Ethics Committee of XXX University (Approval No: 2025/187; Date: 22.04.2025). All procedures were carried out in compliance with the ethical standards of the institutional and national research committees and with the 2013 revision of the Declaration of Helsinki. Given the retrospective nature of the study and the use of anonymized data, the requirement for individual informed consent was formally waived. However, all patients had signed a comprehensive surgical consent form permitting the use of de-identified medical data for research purposes. A completed STROBE checklist for observational cohort studies is provided in the supplementary materials.

### Surgical Technique

All procedures were performed using a standardized laparoscopic sleeve gastrectomy technique under general anesthesia. A 36–40 French bougie was used for gastric calibration in all cases. The greater curvature of the stomach was mobilized starting 4–6 cm from the pylorus and continuing toward the angle of His. Gastric resection was performed using linear staplers, and meticulous hemostasis was ensured along the staple line. No additional bariatric procedures (e.g., hiatal hernia repair, gastric bypass) were performed concurrently. All surgeries were carried out by the same surgical team to ensure technical consistency and reduce procedural variability. Postoperative care included thromboembolism prophylaxis, proton pump inhibitors, and dietary counseling as per institutional protocol [14].

### Data Collection and Variables

Data were retrospectively retrieved from the hospital’s digital patient registry system by two independent researchers to ensure accuracy and reduce potential bias. The collected variables included demographic characteristics such as age and sex; preoperative anthropometric measurements including weight, height, and body mass index (BMI); laboratory parameters consisting of fasting serum triglyceride and fasting blood glucose levels, both expressed in mg/dL; and postoperative outcomes, specifically body weight and BMI recorded at the 12-month follow-up.

TyG index was calculated using a validated and widely accepted formula designed to reflect insulin resistance [15]. Specifically, the TyG index was calculated as ln [fasting triglyceride (mg/dL) × fasting glucose (mg/dL) / 2], integrating two key metabolic parameters into a single continuous variable associated with insulin resistance and metabolic dysfunction.

The primary outcomes of the study were defined as absolute weight loss, measured in kilograms, and percentage weight loss, calculated as the proportion of body weight lost relative to the preoperative baseline.

### Statistical Analysis

All statistical analyses were conducted using IBM SPSS Statistics version 27.0 (IBM Corp., Armonk, NY, USA). The normality of continuous variables was assessed using the Shapiro–Wilk test. Continuous variables were expressed as mean ± standard deviation (SD) or median with interquartile range (IQR), as appropriate. Categorical variables were summarized as frequencies and percentages.

For between-group comparisons, the independent samples t-test was used for normally distributed continuous variables, and the Mann–Whitney U test was applied when normality assumptions were not met. The chi-square test or Fisher’s exact test was used for categorical variables. Pearson correlation analysis was performed to assess linear associations between the TyG index and weight loss outcomes (in both absolute and percentage terms).

To explore the discriminative performance of the TyG index in identifying patients with greater postoperative weight loss, receiver operating characteristic (ROC) curve analysis was performed, and the area under the curve (AUC) was calculated with 95% confidence intervals. The optimal cut-off value for the TyG index was determined using the Youden Index method. Sensitivity and specificity values corresponding to the optimal threshold were also reported to support descriptive interpretation rather than definitive clinical classification.

In order to identify independent predictors of postoperative weight loss, a multivariable linear regression model was constructed. Variables with *p* < 0.10 in univariate analysis or with theoretical relevance (e.g., age, sex, BMI) were included in the final model. Multicollinearity was assessed using variance inflation factor (VIF), and model assumptions were verified via residual analysis.

Prior to data collection, a sample size calculation was conducted using G*Power 3.1 software (Heinrich Heine University, Düsseldorf, Germany). The power analysis was based on an anticipated medium effect size (Cohen’s f² = 0.15), an alpha level of 0.05, and a power of 0.80 for a multiple linear regression model with four predictors. The minimum required sample size was calculated as 75 participants [16]. Therefore, the final sample of 122 patients exceeded this threshold, ensuring adequate statistical power for detecting meaningful associations.

A two-tailed p-value < 0.05 was considered statistically significant for all analyses.

## Results

The study included 122 patients who underwent sleeve gastrectomy. The mean age was 41.2 ± 9.8 years, with 82 females (67.2%) and 40 males (32.8%). The mean preoperative body mass index (BMI) was 46.5 ± 5.4 kg/m². Preoperative triglyceride and fasting glucose levels were 176.3 ± 45.8 mg/dL and 104.7 ± 13.5 mg/dL, respectively. The TyG index was calculated as 8.72 ± 0.51. At the end of the first postoperative year, the mean BMI decreased to 31.4 ± 4.9 kg/m². The average weight loss was 39.3 ± 10.7 kg, and the percentage of weight loss was 28.3 ± 7.4%, as shown in Table 1.

**Table 1:** Baseline demographic and clinical characteristics of the participants (*n* = 122)

Variable	Mean ± SD / *n* (%)
Age (years)	41.2 ± 9.8
Gender (Female/Male)	82 (67.2%) / 40 (32.8%)
Preoperative BMI (kg/m²)	46.5 ± 5.4
Preop Triglyceride (mg/dL)	176.3 ± 45.8
Preop Fasting Glucose (mg/dL)	104.7 ± 13.5
TyG Index	8.72 ± 0.51
Postoperative 1-year BMI (kg/m²)	31.4 ± 4.9
Weight Loss (kg)	39.3 ± 10.7
% Weight Loss	28.3 ± 7.4

Patients were divided into two groups based on the TyG index using a cut-off value of 8.7. When comparing the groups, patients with TyG ≥ 8.7 had a significantly higher mean age (42.8 ± 10.3 vs. 39.6 ± 8.9 years, *p* = 0.043), and a higher preoperative BMI (47.8 ± 5.8 vs. 45.2 ± 4.7, *p* = 0.008). Furthermore, both absolute weight loss (42.5 ± 11.3 kg vs. 36.1 ± 8.9 kg, *p* = 0.001) and percentage weight loss (30.5 ± 7.6% vs. 26.1 ± 6.5%, *p* = 0.002) were significantly greater in the higher TyG group. There was no significant difference in gender distribution (*p* = 0.684), as shown in Table 2.

**Table 2:** Comparison of baseline characteristics and postoperative outcomes according to TyG index group

Variable	TyG < 8.7 (*n* = 61)	TyG ≥ 8.7 (*n* = 61)	*p*-value
Age (years)	39.6 ± 8.9	42.8 ± 10.3	0.043
Gender (F/M)	43 / 18	39 / 22	0.684
Preoperative BMI	45.2 ± 4.7	47.8 ± 5.8	0.008
Weight Loss (kg)	36.1 ± 8.9	42.5 ± 11.3	0.001
% Weight Loss	26.1 ± 6.5	30.5 ± 7.6	0.002

Correlation analysis showed that the TyG index was positively and significantly associated with both absolute weight loss (*r* = 0.42, *p* < 0.05) and percentage weight loss (*r* = 0.38, *p* < 0.05), as shown in Table 3.

**Table 3:** Correlation between TyG index and weight loss outcomes

Variables	Correlation Coefficient (*r*)	*p*-value
TyG vs. Weight Loss (kg)	0.42	< 0.05
TyG vs. % Weight Loss	0.38	< 0.05

Receiver operating characteristic analysis showed that the TyG index exhibited moderate discriminative performance in identifying patients with greater postoperative weight loss, with an area under the curve of 0.73 (95% CI: 0.65–0.81). The optimal cut-off value was 8.7, corresponding to a sensitivity of 75.4% and a specificity of 63.9%. These findings are presented to support descriptive interpretation and were not used for definitive clinical classification.

In multivariable linear regression analysis, the TyG index was found to be an independent predictor of weight loss (B = 2.84, 95% CI: 1.42–4.26, *p* < 0.05). Preoperative BMI was also significantly associated with weight loss (B = 0.41, 95% CI: 0.17–0.65, *p* < 0.05). Age and gender did not reach statistical significance in the model, as shown in Table 4.

**Table 4:** Linear regression analysis of predictors of weight loss

Independent Variable	B Coefficient	95% CI (Lower–Upper)	*p*-value
TyG Index	2.84	1.42–4.26	< 0.05
Age	-0.12	-0.25–0.01	0.067
Gender (Female = 1)	1.09	-1.21–3.39	0.346
Preoperative BMI	0.41	0.17–0.65	< 0.05

## Discussion

This study demonstrated that the preoperative triglyceride-glucose index is significantly and positively associated with postoperative weight loss outcomes in patients undergoing sleeve gastrectomy. The TyG index was found to correlate with both absolute and percentage weight loss, and retained its significance as an independent predictor in multivariable regression analysis. ROC analysis further supported the moderate discriminative performance of the TyG index, identifying an optimal cut-off value of 8.7 with acceptable sensitivity and specificity. Rather than serving as a definitive decision-making tool, these findings suggest that the TyG index may function as a practical, low-cost adjunctive biomarker to support preoperative counseling and metabolic risk stratification in bariatric surgery candidates [17, 18].

The TyG index is a widely recognized surrogate marker for insulin resistance, combining fasting triglyceride and glucose levels—two readily accessible parameters in routine clinical practice. Elevated TyG values reflect a state of disrupted lipid and carbohydrate metabolism. Physiologically, insulin resistance results in increased hepatic gluconeogenesis, reduced peripheral glucose uptake, and enhanced lipolysis, all of which contribute to elevated fasting glucose and triglyceride concentrations. Thus, a high TyG index signifies not just the presence of obesity, but the metabolic dysfunction underlying it [19–21].

Weight loss following sleeve gastrectomy is not solely a result of mechanical gastric restriction. Hormonal and neuroendocrine mechanisms also play a critical role, including reductions in ghrelin levels, increases in GLP-1 and PYY secretion, improved insulin sensitivity, and favorable alterations in gut microbiota [22–24]. Patients with elevated preoperative TyG indices, who are likely to exhibit a greater burden of insulin resistance, may experience more pronounced postoperative metabolic shifts, resulting in enhanced early weight reduction. This concept of “metabolic reactivity” may help explain the observed association between TyG levels and postoperative weight loss outcomes [25].

Our findings are consistent with prior research supporting the use of the TyG index in cardiometabolic risk prediction. Gounden (2024) showed that the TyG index has strong discriminatory power for diagnosing metabolic syndrome [26]. Rivière et al. (2022), in the Collection of Metabolic Tissues study, reported that the TyG index is independently associated with MASLD [27]. Hong et al. (2025) further established its role in identifying cardiometabolic risk across broader clinical populations. The present study extends this literature by exploring the potential relevance of the TyG index in the bariatric surgery setting, particularly with respect to early postoperative weight loss outcomes [28].

Although baseline BMI was also significantly associated with weight loss—as expected given the proportional nature of postoperative weight reduction—the TyG index remained an independent predictor even after adjustment for BMI. This finding suggests that the TyG index provides complementary metabolic information beyond conventional anthropometric measures, capturing aspects of insulin resistance and metabolic health that may influence postoperative weight loss trajectories [29, 30].

One of the strengths of this study is the uniformity of surgical technique, as all procedures were performed by the same surgical team using a standardized laparoscopic sleeve gastrectomy protocol. This minimizes procedural variability and ensures consistency in surgical outcomes. Moreover, the sample size exceeded the minimum required for statistical adequacy, enhancing the reliability of our findings.

From a clinical perspective, the findings of the present study suggest that preoperative metabolic status, as reflected by the TyG index, may contribute to the heterogeneity observed in postoperative weight loss following sleeve gastrectomy. While anthropometric measures such as BMI remain central to patient assessment, they do not fully capture interindividual differences in metabolic responsiveness. In this context, the TyG index may help identify patients who are more likely to exhibit pronounced early metabolic adaptation after surgery, thereby supporting individualized preoperative counseling and expectation management. Importantly, the TyG index should be viewed as a complementary marker rather than a replacement for established clinical parameters, and its potential utility lies in augmenting—not redefining—current preoperative assessment frameworks.

Nevertheless, several limitations should be acknowledged. First, the retrospective and single-center design introduces inherent limitations, including potential selection and information bias, and restricts control over unmeasured confounding variables. Second, detailed preoperative metabolic comorbidities such as type 2 diabetes mellitus and hyperlipidemia were not consistently available for all patients and therefore could not be incorporated into subgroup analyses, which may have provided additional clinical context. Third, other insulin resistance markers, including fasting insulin levels, HOMA-IR, and HbA1c, were not routinely measured and could not be included for comparative validation. Fourth, behavioral and environmental factors known to influence postoperative weight loss—such as dietary adherence, physical activity levels, psychological status, and socioeconomic factors—were not captured in the dataset. Additionally, although all patients were followed for at least 12 months, long-term outcomes such as weight regain, durability of weight loss, and resolution of associated medical problems could not be assessed. Finally, the identified TyG cut-off value should be interpreted as exploratory and requires prospective, multicenter validation before broader clinical application.

Despite these limitations, this study provides novel insight into the potential clinical relevance of the TyG index in the bariatric surgery setting. The simplicity, reproducibility, and low cost of the TyG index make it an attractive adjunct to routine preoperative assessment, particularly for enhancing metabolic profiling and informing patient counseling. Future prospective studies with longer follow-up and external validation cohorts are warranted to clarify the long-term prognostic value and clinical applicability of TyG-based risk stratification in bariatric populations.

## Conclusion

In conclusion, the preoperative triglyceride-glucose index was found to be significantly associated with postoperative weight loss in patients undergoing sleeve gastrectomy and remained independently associated with early postoperative weight loss outcomes after adjustment for key clinical variables. This simple, inexpensive, and easily accessible biomarker may serve as a valuable adjunctive tool for preoperative risk stratification and patient counseling. By reflecting underlying metabolic dysfunction, the TyG index provides information beyond conventional anthropometric measures such as BMI. Rather than guiding surgical eligibility, integrating the TyG index into routine preoperative assessment may enhance metabolic profiling and support more individualized counseling and expectation management. Further large-scale, prospective studies are warranted to confirm these findings, validate clinically meaningful cut-off values, and to explore the long-term prognostic value of the TyG index in bariatric populations.

## Data Availability

All data and materials can be requested from Dr. Muhammet Fatih Keyif when needed. (drfatihkeyif16@gmail.com).
